# Changes in health behaviours in adults at-risk of chronic disease: primary outcomes from the *My health for life* program

**DOI:** 10.1186/s12889-022-14056-1

**Published:** 2022-08-30

**Authors:** Charrlotte Seib, Stephanie Moriarty, Nicole McDonald, Debra Anderson, Joy Parkinson

**Affiliations:** 1 School of Nursing and Midwifery, Griffith University, Queensland, Australia; 2 Menzies Health Institute of Queensland, Griffith University, Queensland, Australia; 3 Australian eHealth Research Centre, CSIRO, Queensland, Australia; 4 Institute of Urban Indigenous Health, Brisbane, Australia; 5 Faculty of Health, University of Technology Sydney, New South Wales, Australia

**Keywords:** Healthy lifestyle index, Chronic disease prevention, Health promotion, Health behaviour change, Dietary intake, Body mass index, Waist circumference, Smoking, Physical activity

## Abstract

**Background:**

Chronic disease is the leading cause of premature death globally, and many of these deaths are preventable by modifying some key behavioural and metabolic risk factors. This study examines changes in health behaviours among men and women at risk of diabetes or cardiovascular disease (CVD) who participated in a 6-month lifestyle intervention called the *My health for life* program.

**Methods:**

The *My health for life* program is a Queensland Government-funded multi-component program designed to reduce chronic disease risk factors amongst at-risk adults in Queensland, Australia. The intervention comprises six sessions over a 6-month period, delivered by a trained facilitator or telephone health coach. The analysis presented in this paper stems from 9,372 participants who participated in the program between July 2017 and December 2019. Primary outcomes included fruit and vegetable intake, consumption of sugar-sweetened drinks and take-away, alcohol consumption, tobacco smoking, and physical activity. Variables were summed to form a single Healthy Lifestyle Index (HLI) ranging from 0 to 13, with higher scores denoting healthier behaviours. Longitudinal associations between lifestyle indices, program characteristics and socio-demographic characteristics were assessed using Gaussian Generalized Estimating Equations (GEE) models with an identity link and robust standard errors.

**Results:**

Improvements in HLI scores were noted between baseline (Md = 8.8; IQR = 7.0, 10.0) and 26-weeks (Md = 10.0; IQR = 9.0, 11.0) which corresponded with increases in fruit and vegetable consumption and decreases in takeaway frequency (*p* < .001 for all) but not risky alcohol intake. Modelling showed higher average HLI among those aged 45 or older (β = 1.00, 95% CI = 0.90, 1.10, *p* < .001) with vocational educational qualifications (certificate/diploma: β = 0.32, 95% CI = 0.14, 0.50, *p* < .001; bachelor/post-graduate degree β = 0.79, 95% CI = 0.61, 0.98, *p* < .001) while being male, Aboriginal or Torres Strait Islander background, or not currently working conferred lower average HLI scores (*p* < .001 for all).

**Conclusions:**

While participants showed improvements in dietary indicators, changes in alcohol consumption and physical activity were less amenable to the program. Additional research is needed to help understand the multi-level barriers and facilitators of behaviour change in this context to further tailor the intervention for priority groups.

**Supplementary Information:**

The online version contains supplementary material available at 10.1186/s12889-022-14056-1.

## Background

Chronic disease poses the greatest threat to global health, with a higher morbidity and mortality rate, than do all other causes contributing to around 41 million deaths each year [1]. Currently, chronic diseases- namely cardiovascular diseases, cancer, diabetes, and chronic respiratory diseases account for over 80% of all premature chronic disease deaths [1]. The prevalence in Australia is similar with more than three-quarters of all deaths in 2018 attributable to one of several major chronic conditions (cardiovascular disease, cancer, chronic obstructive pulmonary disease, diabetes, asthma, chronic kidney disease, and mental illness) and a further 47% of Australian adults are living with at least one chronic condition [2]. The ramifications of chronic disease burden to individuals, their families, and the wider community is significant, collectively costing the Australian economy between $840 million and $185 billion annually [2–4].

The development of chronic disease is underpinned by varying risk factors, including both non-modifiable (e.g., age, sex, ethnicity) and modifiable health behaviours [1–3, 5] with behavioural and metabolic risk factors accounting for 45.8% of global disease burden in 2015 (30.3% behavioural and 15·5% metabolic) [6]. During the same period, an estimated 38% of total burden of disease experienced by Australians was attributable to tobacco use, overweight and obesity, dietary risks, hypertension, and hyperglycaemia [3]. Clearly, modifiable health behaviours including smoking, poor nutrition, excessive alcohol consumption and insufficient physical activity pose a significant public health issue in high-, medium-, and low- income countries alike, and there is an urgent need for action [7].

Addressing chronic disease is important for the 2030 Agenda for Sustainable Development, specifically Sustainable Development Goal (SDG) target 3.4 calls for a one-third reduction in premature mortality from chronic disease by 2030 [7]. To accelerate progress in attaining SDGs and reduce the risk of chronic disease, deliberately designed interventions targeting smoking cessation, reduction of harmful alcohol use, healthy eating and increased physical activity are needed [8–10]. Well-developed health promotion interventions are cost-effective and sustainable in improving population health and reducing risks for chronic disease [11]. The need for health promotion programs is compelling, with the complexity of current threats to health and wellbeing, with the most disadvantaged in society bearing the greatest burden, means there is a need for approaches which account for complex, concurrent risk factors. Comprehensive approaches, co-created with participants and that account for the interplay between risk factors have potential to bring about the scale and scope of changes needed for sustainable health improvement at the population level [12]. For example, a recent study of 304,779 adolescents from 89 countries showed clustering between modifiable health behaviours of physical inactivity and inadequate fruit and vegetable intake and the co-occurrence of tobacco smoking, alcohol drinking, physical inactivity, and poor dietary indicators (though this effect was stronger in females than males) [13].

Modifiable health behaviours including diet, alcohol, tobacco smoking, and physical activity are linked with physical and psychological symptoms including pain, fatigue and depressive symptoms [14]. Among people participating in community lifestyle programs, positive clinical and behavioural outcomes are often associated with corresponding improvements in general health [15, 16]. Building on the success of multiple health behaviour approaches to disease prevention in other Australian locales (see for example https://www.lifeprogram.org.au/), the Queensland Government invested in a large public health program, *My health for life.* The program, aims to reduce the risk of cardiovascular disease and diabetes in priority groups, such as those at high risk of developing chronic disease, Aboriginal and Torres Strait Islander People and culturally and linguistically diverse people, through supporting individuals to make changes to their health behaviours. The program targets multiple modifiable health behaviours associated with increased chronic disease risk, therefore to assess the joint association of the multiple modifiable health behaviours targeted by the program, a healthy lifestyle index (HLI) score was created by combining dietary, alcohol and tobacco smoking, and physical activity indices to form a composite score. To understand the effectiveness of the program, the purpose of this paper is to examine changes in the primary outcome (health behaviour measured using the HLI score) in participants of the *My health for life* program.

## Methods

The *My health for life* program is a novel, multi-component program that aimed to reduce chronic disease risk factors among adults at risk of diabetes or CVD in the state of Queensland, Australia. The Queensland Government funded program was developed by an alliance of health organisations led by Diabetes Queensland including Stroke Foundation, Heart Foundation, Queensland Primary Health Networks, Ethnic Communities Council of Queensland and Queensland Aboriginal and Islander Health Council (collectively referred to as the Healthier Queensland Alliance). Details about the program are here https://www.myhealthforlife.com.au/.

To recruit people at-risk of developing chronic disease, Stroke Foundation staff undertook health checks at community events and workplaces across the state of Queensland, health clinicians undertook health checks in clinical settings (e.g., allied health clinic, pharmacy or general practice) and potential participants undertook a health check online via the website (https://www.myhealthforlife.com.au/risk-assessment). A range of marketing and communication activities were undertaken to lead potential participants to the online health check including television advertisements on regional and metropolitan television channels, outdoor advertising on billboards and bus shelters, Facebook advertising and print advertisements in local newspapers and a motoring club magazine. Eligible participants were identified using adapted risk assessment tools (stemming from Australian Diabetes Risk assessment (AUSDRISK) [17] or Absolute Cardiovascular Disease Risk assessment (CVD Check) [18]). The program was offered to eligible ‘high risk’ adults aged 45 years and over (or 18 years for Aboriginal and/or Torres Strait Islander peoples due to their increased risk of developing chronic disease) [19]. High risk of chronic disease was determined by an adapted AUSDRISK Assessment score ≥ 12, Absolute Cardiovascular Disease risk ≥ 15% or blood pressure reading ≥ 160 mmHg over ≥ 100 mmHg.

The program offered is either the face-to-face group-based program (GBP) or one-on-one telephone health coaching (THC), with participants choosing the most suitable option for themselves. The GBP consists of small groups of 6–8 participants, delivered by a trained facilitator in a community setting running for approximately two hours and the THC offering is delivered one-on-one via telephone in house by the lead organisation, Diabetes Queensland with a trained facilitator (telephone health coach) running for approximately one hour. Potential provider organisations for the face-to-face program responded to a call for expressions of interest to deliver the program. The organisations nominated qualified and experienced health professionals for training by the program implementation team as a facilitator for the delivery of the face-to-face program with 136 approved providers engaged to deliver the program. Diabetes Queensland recruited the telephone health coaches to work in house through a standard employment recruitment process of qualified and experienced health professionals or through identification of appropriately qualified and experienced existing health professional staff. In total, 408 health professionals attended facilitator training with 403 completing training to become a certified facilitator. Of these, 389 were trained and certified to deliver face-to-face groups, and 14 were trained and certified to deliver the THC program. Training of facilitators included completion of prior reading, attendance at a two day face-to-face training course and successful completion of all assessment. All facilitators are required to maintain accreditation through participation in professional development activities on an annual basis. Training and certification of facilitators was conducted be the My health for life program implementation team. Most facilitators were contracted to an Allied Health Service (*n* = 245, 63.0%), with a small number contracted to a Pharmacy (*n* = 6, 1.5%). In total, 264 facilitators (face-to-face or THC) delivered at least one program and had a variety of backgrounds in Allied Health (Dietetics or Exercise Physiology), Nursing, Pharmacy, Health Promotion, Counselling, Aboriginal Health Work or Multicultural Health Work. Retention of facilitators that delivered at least one program was 61.7% (*n* = 163). Retention of provider organisations that delivered at least one program was 81.8% (*n* = 112). All provider organisations received financial payment to deliver the face-to-face program, paid on a per participant basis.

Both the GBP and THC program comprise six sessions over a 6-month period at fortnightly intervals (sessions 1–5) with session 6 (related to maintenance) occurring at around 24 weeks. The program, underpinned by the Health Action Process Approach (HAPA), aimed to develop knowledge, skills, and strategies to adopt positive lifestyle behaviours, while educating participants on different risk factors, including healthy eating, alcohol, tobacco use, and physical activity. Session activities target modifiable health behaviours using behaviour change techniques [20] as outlined in Table 1. HAPA, chosen as it targets self-efficacy and coping and has behaviour change techniques [21] embedded, is a dynamic model with a motivational phase, followed by a volitional phase appropriate for a six-session behaviour change program. Program delivery is supported by a workbook and program manual for participants in both the GBP and THC program.

**Table 1:** Program activities and behaviour change techniques

Session	Timeline	Activity	Behaviour change technique
1	Week 1 (Survey 1)	Introduction to the program Set your intention	Motivational interview Intention formation
2	Week 3	Understanding risk factors and preventing chronic diseases Find the why- discovering motivation	Barrier identification
3	Week 5	Physical activity guidelines Goal setting	Specific goal setting
4	Week 7	Healthy eating guidelines Engaging support	Planning social support
5	Week 9 (Survey 2)	Alcohol and smoking guidelines Adjusting for changes	Review of behavioural goals Time management Relapse prevention
6	Week 21 (Survey 3)	Maintaining healthy habits Preventing relapse	Relapse prevention

This paper draws upon survey data from 9,372 participants of the *My Health for Life* program between July 2017 and December 2019 who contributed weight, diet, alcohol, smoking and physical activity data towards the composite healthy lifestyle index (HLI). Participants consented to participate upon commencement in the program and completion of the first survey. Telephone health coaches or program facilitators assisted participants to enrol in the program. Ethical approval was granted from the Darling Downs Health Human Research Ethics Committee (HREA/2021/QTDD/72406) and Griffith University (GU Ref No: 2021/143) before accessing de-identified, secondary data.

### Measurements

This study uses a pragmatic non-randomised, time–series analysis adopting observational, goal-based and pretest–posttest design for the program evaluation (see [22] for full details of evaluation). Data were collected during sessions at three timepoints, session 1 (week 1), session 5 (week 12), and session 6 (week 24) via either a self-administered paper survey (GBP participants) or interviewer-administered with data directly entered into the online data portal (THC participants). Facilitators assisted GBP participants to complete the survey, taking waist measurements and weight using supplied scales. THC participants used their own measurement equipment, however, were guided through the process by their telephone health coach. Facilitators and telephone health coaches provided guidance on what serves of fruit and vegetables look like and this was also written in the paper surveys (written and verbal guidance for vegetables provided was, “a serve is half a cup of cooked vegetables or one cup of salad vegetables”. For fruit was, “a serve is one medium piece or two small pieces of fruit or a cup of diced pieces”). Telephone health coaches, to ensure consistency of data entry, then entered the paper survey data into the online data portal. Primary outcome variables included fruit and vegetable intake, consumption of sugar-sweetened drinks and take-away, alcohol and tobacco smoking, and physical activity.

#### Diet

Four items from the General Population Health Survey [23] comprised the dietary indicator. They included daily serves of fruit and vegetables (none/less than 1 serve/1- 5 serves/6 or more serves), sugar-sweetened drinks (daily/several times per week/about once a week/about once a fortnight/about once a month/less often than once per month/never) and takeaway consumption (everyday/weekly/monthly/rarely/never) which were grouped according to Australian Dietary Guidelines [24]. Healthy diet was defined as two or more serves of fruit, five or more serves of vegetables, infrequent sugar-sweetened drinks (either weekly or less than weekly) and take-away (either weekly or less than weekly) consumption.

#### Alcohol and tobacco smoking

Alcohol and tobacco smoking were measured using 3-items from the Australian Health Survey [25]. Alcohol use was grouped according to the *2009 National Health and Medical Research Council* [26] with healthy alcohol intake measured as ≤ 4 drinks per session and consuming alcohol less than weekly (and not daily). Tobacco smoking, measured using one item, grouped as current smoker, former smoker or never smoked. Alcohol was measured using 2 items; quantity (number of standard drinks consumed in a single session, range < 1 – > 20) and frequency (daily/weekly/monthly/rarely/never).

#### Physical activity

The physical activity indicator was measured using a single item “What do you estimate was the total time you spent doing physical activities in the last week? Please answer in minutes, for example if you did a total of one hour then write 60 min”, obtained from the Active Australia Survey [27]. The variable, collapsed to form a single trichotomous variable indicating whether individuals were sufficiently active for health, insufficiently active, or sedentary. Sufficient activity for health, was categorised as 30 min of physical activity on at least 5 days of the week with a total of at least 150 min of activity per week. Insufficient activity was categorised as some physical activity, but not in sufficient frequency or duration to obtain a health benefit. Sedentary lifestyle was categorised as an absence of all physical activity [27].

#### Healthy Lifestyle Index

The healthy lifestyle index was derived from current Australian guidelines for good health [23–27]. Initially, common lifestyle factors for diet, alcohol and tobacco smoking, and physical activity were combined to form single scores before an overall composite score was computed.

Diet was defined using 4 indicators including the minimum daily serves of fruit (0 =  < 2 serves, 1 =  ≥ 2 serves) and vegetables (0 =  < 5 serves, 1 =  ≥ 5 serves), intake of sugar-sweetened drinks (0 =  > weekly, 1 = weekly, 2 =  < weekly) and take-away consumption (0 = daily, 1 = weekly, 2 =  < weekly) [24]. The dietary index was computed as the sum of all four indicators (range 0 – 6) with higher scores representing greater compliance with dietary guidelines.

The alcohol and tobacco index, based on the health guidelines for drinking alcohol [26], comprised 3 indictors outlining alcohol frequency (0 = daily, 1 = less than daily), alcohol quantity (2 = none, 1 = 1–4 drinks per session, 3 =  ≥ 5 drinks per session), and smoking status (0 = current smoker, 1 = former smoker, 2 = never smoked). The final index was computed by summing the 3 indicators with higher scores denoting less alcohol and smoking (range 0 – 5).

For the physical activity component, a single indicator was used. The variable, derived from the Active Australia Survey, was collapsed to form a single trichotomous variable indicating being sedentary (no points), insufficiently active (1 point), and sufficiently active for health (2 points) [27].

Details of the scoring for each indicator is in Supplementary Table 1. To create the HLI, the dietary, alcohol, smoking, and physical activity indexes were summed using a simple additive method.^1^ The final score ranged from 0 to 13, with higher scores denoting a healthier diet (≥ 2 serves of fruit and 5 serves of vegetables and infrequent consumption of sugar-sweetened drinks and take-away food), abstinence from alcohol and cigarette smoking, and higher physical activity (least 150 min of activity over one week.

#### Covariates

Overweight and obesity are associated with around 8% of Australia’s burden of disease [3] and was thus, one of the targeted health behaviours for the *My health for life program*. However, while excess weight was a primary outcome for the study, it was not included in the healthy lifestyle index as it could have been an intermediate factor between modifiable health behaviour and health outcomes [28]. Nevertheless, we included baseline body mass index (BMI) and waist circumference (WC) in a sensitivity analysis (see in Supplementary Table 2). In this study, BMI was grouped according to adult weight guidelines [29] with a BMI < 25 kg/m^2^ representing normal weight, 25–29.9 kg/m^2^ representing overweight, 30–39.9 kg/m^2^ representing obesity and >  = 40 kg/m^2^ representing extreme obesity. Sex-specific waist circumference was grouped according to increased risk (94-101 cm in men and 80-87 cm in women) and greater increased risk (> 102 cm in men and > 88 cm in women). Both measures were included in this analysis to adequately capture adiposity. BMI is an adequate measure of adiposity for clinical purposes [30] whereas among overweight/class-I obese (i.e., BMI 25—34.9 kg/m2) individuals, waist circumference is preferred as it provides additional information about increased disease risk [31].

Adjustment was made for other covariates including socio-demographic characteristics (i.e., sex, socio-economic status, ethnicity, education, First Nations People (i.e., Aboriginal and/or Torres Strait Islander background), Culturally or Linguistically Diverse (CALD) background, and employment [32]), relative socio-economic advantage and disadvantage (derived from the Australian Bureau of Statistics Index of Relative Socio-Economic Advantage and Disadvantage (IRSAD) that compares the relative economic and social conditions of people and households within a specific geographic area [13]), and study variables (modality: THC vs. GBP; number of sessions attended, range 1–6).

### Data analysis

Statistical analyses were performed using SPSS (Statistical Package for the Social Sciences) version 23 [33] and STATA 13 [34]. Descriptive data are expressed as counts and percentages, mean, and standard deviation (SD), and bivariate statistics were performed using chi-square (χ^2^) tests and ANOVA with statistical significance set at α = 0.01 and clinical significance achieved with percentage differences greater than 10% [35].

Before undertaking multivariate analysis, the patterns of missing data were examined. For the primary outcomes, the amount of missing data at Session 1 varied from < 1% on dietary and alcohol indicators (smoking, 1.9%; physical activity, 7.1%) (see Supplementary Tables 3 and 4 for additional detail). Analysis of the missing patterns showed Session 1 missingness was strongly correlated with program modality (94% occurred in THC participants) and several participant socio-demographic characteristics (see Supplementary Table 3), and so data were not plausibly missing completely at random.

However, while data were not missing completely at random, the missing data comprised less than 10%, not perceived to bias results [36–38]. Thus, multiple imputation by monotone conditional univariate equations were performed using the ‘regress’ command in Stata [39]. All analysis and auxiliary variables were included in the imputation model to improve the prediction of missing values [36] with fifty imputed datasets generated [16]. To assess the robustness of the multiply imputed data parameter estimates, data for the observed sample were presented alongside the imputed data at each timepoint (Sessions 1, 5 and 6).

Longitudinal associations between lifestyle indices were assessed using GEE models with an identity link and robust standard errors [40–42]. GEE was chosen for is ability to deal with longitudinal and clustered data. To determine the best working correlation matrix, the Quasi-likelihood under the Independence model Criterion (QIC) was computed with the an exchangeable correlation structure best fitting the data [43, 44]. Separate models were fitted for HLI estimates for time only (Model 1), for time and program characteristics (study modality and number of sessions attended; Model 2), and for time, program characteristics and personal background (employment status, sex, age bracket, educational attainment, First Nations People, and IRSAD quintile; Model 3). Finally, to assess the contribution of individual dietary, alcohol and smoking, and physical activity indices, a lasagne (or lasagna) plot was generated [45, 46] using the predicted probabilities from nominal logistic models that were fitted for each health behaviour separately while adjusting for study modality, number of sessions attended, time, employment status, sex, age bracket, educational attainment, First Nations People, and IRSAD quintile.

## Results

This paper presents primary outcome data from 9,372 Queensland adults who participated in the *My health for life* program from July 2017 to December 2019. Tables 2 and 3 presents baseline study modality, and socio-demographic characteristics by healthy lifestyle indices grouped into quintiles (Quintile 1 represents unhealthy lifestyle behaviours; Quintile 5 represents greatest number of healthy lifestyle behaviours). The study sample of First Nations People (Aboriginal and Torres Strait Islander people) (4.1%) is slightly higher than in the Australian population (3.3%). There is under representation in the lower IRSAD quintiles (Q1 = 13%, Q2 = 16.3%) and over representation in higher quintiles (Q4 = 22.3% and Q5 = 25.6%), which is to be expected given these participants may be more motivated to improve their health behaviours. There are higher levels of female participants (77.3%) included in this study. Education level in the study sample was slightly higher for Bachelor degree or postgraduate degree (28.8%) compared to the Australian population (25.8%), and for certificate or diploma (36.2%) compared to Australian population (26.1%), and similar for primary school education (3.4%) compared to Australian population (4.4%).

**Table 2:** Baseline characteristics by healthy lifestyle index (HLI) quintiles ^a^

	Quintile 1	Quintile 2	Quintile 3	Quintile 4	Quintile 5	Total
	n (%)	n (%)	n (%)	n (%)	n (%)	n (%)
Mode						
THC	599 (38.5)	1,079 (39.6)		481 (30.6)	504 (29.8)	3,339 (35.6)*
GBP	958 (61.5)	1,647 (60.4)		1,089 (69.4)	1,190 (70.2)	6,033 (64.4)
Employment status						
Employed	937 (63.4)	1,503 (57.7)	912 (52.2)	756 (50.5)	739 (45.5)	4,847 (54.1)*
Home duties	93 (6.3)	135 (5.2)	89 (5.1)	56 (3.7)	55 (3.4)	428 (4.8)
Retired	164 (11.1)	576 (22.1)	536 (30.7)	533 (35.6)	688 (42.3)	2,497 (27.9)
Not working	163 (11.0)	220 (8.4)	103 (5.9)	81 (5.4)	60 (3.7)	627 (7.0)
Other	120 (8.1)	171 (6.6)	108 (6.2)	72 (4.8)	83 (5.1)	554 (6.2)
Gender						
Female	1,091 (70.6)	2,017 (74.5)	1,451 (79.8)	1,275 (81.7)	1,372 (81.4)	7,206 (77.3)*
Male	455 (29.4)	691 (25.5)	368 (20.2)	285 (18.3)	313 (18.6)	2,112 (22.7)
Age bracket						
< 45 years	459 (29.6)	457 (16.8)	211 (11.6)	132 (8.4)	103 (6.1)	1,362 (14.6)*
45 or older	1,092 (70.4)	2,262 (83.2)	1,612 (88.4)	1,435 (91.6)	1,588 (93.9)	7,989 (85.4)
First Nations People						
No	1,417 (91.0)	2,611 (95.8)	1,760 (96.4)	1,540 (98.1)	1,657 (97.8)	8,985 (95.9)*
Yes	140 (9.0)	115 (4.2)	65 (3.6)	30 (1.9)	37 (2.2)	387 (4.1)
Educational attainment						
Primary education	55 (3.6)	102 (3.8)	62 (3.5)	44 (2.9)	49 (3.0)	312 (3.4)*
Secondary education	499 (32.8)	812 (30.3)	548 (30.6)	437 (28.4)	464 (28.1)	2,760 (30.1)
Certificate/diploma	647 (42.5)	973 (36.3)	621 (34.7)	524 (34.1)	561 (33.9)	3,326 (36.2)
Bachelor/postgraduate	300 (19.7)	754 (28.1)	536 (30.0)	499 (32.5)	554 (33.5)	2,643 (28.8)
Other	21 (1.4)	40 (1.5)	22 (1.2)	33 (2.1)	25 (1.5)	141 (1.5)
CALD						
No	1,513 (97.2)	2,637 (96.7)	1,776 (97.3)	1,516 (96.6)	1,654 (97.6)	9,096 (97.1)
Yes	44 (2.8)	89 (3.3)	49 (2.7)	54 (3.4)	40 (2.4)	276 (2.9)
IRSAD quintile						
Quintile 1 *(most advantaged)*	245 (15.8)	371 (13.6)	231 (12.7)	182 (11.6)	189 (11.2)	1,218 (13.0)*
Quintile 2	320 (20.6)	497 (18.2)	265 (14.5)	222 (14.1)	221 (13.1)	1,525 (16.3)
Quintile 3	367 (23.6)	587 (21.5)	434 (23.8)	349 (22.2)	399 (23.6)	2,136 (22.8)
Quintile 4	316 (20.3)	607 (22.3)	406 (22.3)	355 (22.6)	406 (24.0)	2,090 (22.3)
Quintile 5 *(most disadvantaged)*	307 (19.7)	664 (24.4)	486 (26.7)	461 (29.4)	477 (28.2)	2,395 (25.6)
General health						
Fair/poor	928 (60.8)	1,231 (45.8)	665 (36.7)	496 (31.9)	356 (21.1)	3,676 (39.7)*
Excellent/good	598 (39.2)	1,459 (54.2)	1,145 (63.3)	1,057 (68.1)	1,328 (78.9)	5,587 (60.3)
Frequent mental distress^b^						
No	967 (66.0)	1,840 (72.0)	1,302 (76.8)	1,179 (80.9)	1,358 (84.5)	6,646 (75.7)*
Yes	499 (34.0)	716 (28.0)	393 (23.2)	279 (19.1)	249 (15.5)	2,136 (24.3)
Frequent unhealthy days^b^						
No	691 (49.0)	1,333 (54.5)	1,011 (61.5)	898 (64.6)	1,112 (71.5)	5,045 (59.7)*
Yes	720 (51.0)	1,115 (45.5)	633 (38.5)	492 (35.4)	443 (28.5)	3,403 (40.3)

*THC* Telephone health couching, *GBP* Group-based program, *CALD* Culturally or Linguistically Diverse, *IRSAD* Index of Relative Socio-economic Advantage and Disadvantage

^a^ Highest quintile represents greatest number of healthy lifestyle indices while the lowest represents most unhealthy lifestyle behaviours

^b^ Frequent unhealthy day and frequent mental distress is defined as 14 or more days of the past 30 day [4, 5]

^*^p < .01

**Table 3:** Percentage of healthy behaviours among complete cases at Sessions 1, 5 and 6

	Session 1	Session 5	Session 6
	n (%)	n (%)	n (%)
Diet index			
Daily fruit intake^a^			
< 2 serves	5,032 (53.7)	1,716 (29.2)	1,097 (26.5)*
2 or more serves	4,340 (46.3)	4,168 (70.8)	3,047 (73.5)
Daily veg. intake^a^			
< 5 serves	8,447 (90.1)	4,522 (76.8)	3,081 (74.3)*
5 or more serves	925 (9.9)	1,363 (23.2)	1,064 (25.7)
Sugar-sweetened drinks			
More than weekly	1,531 (16.3)	559 (9.1)	410 (8.2)*
Once a week	1,071 (11.4)	715 (11.6)	497 (9.9)
Less than weekly	6,770 (72.2)	4,869 (79.3)	4,103 (81.9)
Takeaway			
More than weekly	29 (0.3)	14 (0.2)	6 (0.1)*
Once a week	3,289 (35.1)	1,416 (23.0)	993 (19.8)
Less than weekly	6,054 (64.6)	4,726 (76.8)	4,014 (80.1)
Alcohol and smoking index			
Alcohol quantity			
5 or more	3,574 (38.1)	2,371 (38.5)	1,913 (38.2)*
1–4 drinks	1,584 (16.9)	943 (15.3)	650 (13.0)
None	4,214 (45.0)	2,840 (46.1)	2,450 (48.9)
Alcohol frequency			
Daily	203 (2.2)	58 (0.9)	45 (0.9)*
Weekly or less	9,169 (97.8)	6,096 (99.1)	4,967 (99.1)
Smoking status			
Current	752 (8.0)	348 (3.9)	301 (3.3)*
Former	2,248 (24.0)	2,334 (25.9)	2,350 (26.1)
Never	6,372 (68.0)	6,339 (70.3)	6,362 (70.6)
Physical activity index			
Physical activity^b^			
Sedentary	1,803 (19.2)	383 (6.5)	549 (10.9)*
Insufficient for health	4,370 (46.6)	2,104 (35.5)	1,802 (35.8)
Sufficient for health	3,199 (34.1)	3,433 (58.0)	2,687 (53.3)

^a^Current dietary guidelines recommend a minimum of 2 fruit per day and 5 serves of vegetables [25]

^b^Physical activity was defined according to the Australian Physical Activity Guidelines [26] denoting the accumulation of at least 150 min of activity over one week

^*^ *p* < .01

Baseline bivariate comparisons of the healthy lifestyle index showed that healthy lifestyle was associated with age (45 years or older; *χ*^*2*^(4) = 285.15, *p* < 0.01), sex (female; *χ*^*2*^(4) = 22.34, *p* < 0.001), retirement (*χ*^*2*^(16) = 328.41, *p* < 0.001), higher educational attainment (*χ*^*2*^(16) = 79.10, *p* < 0.001), and greater relative advantage (IRSAD Quintiles 4 and 5; χ2(16) = 124.93, *p* < 0.001). Socio-demographic characteristics by HLI quintile are further outlined in Table 2.

Overall, three-quarters of participants were female, most were aged 45 years or older (> 80%), around two-thirds reported a secondary school or certificate/diploma level education, and half were employed outside the home. Some modest but statistically significant differences were noted with attrition highest in men (*χ*^*2*^(4) = 16.41, *p* < 0.01) aged 45 years or less (*χ*^*2*^(2) = 67.36, *p* < 0.01) with primary or secondary school education (*χ*^*2*^(8) = 16.93, *p* = 0.03).

Table 4 presents the descriptive health behaviours for complete cases at Sessions 1, 5 and 6. The proportion of participants consuming recommended daily serves of fruit (Session 1, 46.3%; Session 5, 70.8%; Session 6, 73.5%, *p* < 0.001) and vegetables increased over time (Session 1, 9.9%; Session 5, 23.2%; Session 6, 25.7%, *p* < 0.001) while the frequency takeaways decreased. Risky alcohol intake (i.e., daily drinking or having more than 4 standard drinks on any one day [25]) was largely unchanged over the program period though current cigarette smoking decreased from 8.0% at Session 1 to 3.3% at Session 6 (*p* < 0.01 but percentage differences < 10% [35]). Finally, the proportion of participants who were sufficiently active for health according to the Australian Physical Activity Guidelines [27] increased from 34.1% at Session 1 to 53.3% at Session 6.

**Table 4:** Summary statistics for the original and imputed healthy lifestyle indices

	Session 1		Session 5		Session 6	
	Original	Imputed	Original	Imputed	Original	Imputed
Healthy lifestyle index^a^						
n	5858	213,454	3928	134,991	2568	138,405
M(SD)	8.6 (2.1)	8.4 (2.0)	9.6 (1.9)	9.6 (1.9)	9.7 (1.8)	9.9 (1.9)
Median [IQR]	9.0 [7.0, 10.0]	8.8 [7.0, 10.0]	10.0 [8.0, 11.0]	10.0 [8.1, 11.0]	10.0 [9.0, 11.0]	10.0 [9.0, 11.0]
Minimum	1	1	3	0	2	2
Maximum	13	17	13	17	13	18

^a^Healthy lifestyle index computed as the sum of dietary, physical activity and alcohol and smoking

However, while there were general trends towards healthy lifestyle behaviours over the program period, attrition might have influenced prevalence and therefore data were imputed. To assess the robustness of imputation, the original and imputed healthy lifestyle indices summary statistics are provided. Point estimates for the HLI (range 0—13) did not change at each time point with the average HLI at Session 1 being 8.6 (SD = 2.1), 9.6 (SD = 1.9) at Session 5 and 9.9 (SD = 1.9) at Session 6.

The results of Gaussian Generalized Estimating Equations which incrementally adjusted for program characteristics (Model 2) and personal background (Model 3) are shown in Table 5. Over the program period, the average HLI increased by around 1-point at Session 5 (Model 1: β = 0.97, 95% CI = 0.90, 1.03, *p* < 0.001; Model 2: β = 0.96, 95% CI = 0.89, 1.03, *p* < 0.001; Model 3: β = 0.98, 95% CI = 0.91, 1.05, *p* < 0.001) and this was sustained at Session 6 (Model 1: β = 1.20, 95% CI = 1.13, 1.27, *p* < 0.001; Model 2: β = 1.19, 95% CI = 1.12, 1.27, *p* < 0.001; Model 3 β = 1.20, 95% CI = 1.13, 1.28, *p* < 0.001).

**Table 5:** Longitudinal modelling of a HLI using GEE with an exchangeable structure and robust standard errors

	Model 1^a^		Model 2^b^	Model 3^c^
	*β (95% CI)*		*β (95% CI)*	*β (95% CI)*
Constant	8.51 (8.46, 8.55)*		8.07 (7.97, 8.17)*	6.85 (6.63, 7.08)*
Sessions				
Session 1	-		-	-
Session 5	0.97 (0.90, 1.03)*		0.96 (0.89, 1.03)*	0.98 (0.91, 1.05)
Session 6	1.20 (1.13, 1.27)*		1.19 (1.12, 1.27)*	1.20 (1.13, 1.28)
Mode				
THC			-	-
GBP			0.14 (0.07, 0.21)*	0.14 (0.07, 0.21)
No. sessions (range 1–6)			0.10 (0.07, 0.12)*	0.05 (0.03, 0.08)*
Employment status				
Employed				-
Home duties				-0.08 (-0.23, 0.06)
Retired				0.59 (0.51, 0.66)*
Not working				-0.46 (-0.61, -0.32)*
Other				-0.09 (-0.24, 0.06)
Sex				
Female				-
Male				-0.49 (-0.57, -0.42)*
Age bracket				
< 45 years				-
45 or older				1.00 (0.90, 1.10)*
Educational attainment				
Primary education				-
Secondary education				0.20 (0.01, 0.38)
Certificate/diploma				0.32 (0.14, 0.50)*
Bachelor/postgraduate				0.79 (0.61, 0.98)*
Other				0.53 (0.24, 0.82)*
First Nations People				
No				-
Yes				-0.49 (-0.68, -0.30)*
IRSAD quintile				
Quintile 1				-
Quintile 2				-0.04 (-0.16, 0.08)
Quintile 3				0.18 (0.07, 0.29)*
Quintile 4				0.22 (0.11, 0.33)*
Quintile 5				0.34 (0.23, 0.45)*

*THC* Telephone health couching, *GBP* Group-based program, *IRSAD* Index of Relative Socio-economic Advantage and Disadvantage

^a^Model 1, unadjusted relationship between HLI and time (sessions 5 and 6)

^b^Model 2, adjusted for program characteristics (delivery mode and no. sessions attended)

^c^Model 3, adjusted for program characteristics and personal background (employment status, sex, age bracket, educational attainment, First Nations People, and IRSAD quintile)

^*^ *p* < .01

Model 2 examined the additive effect of program characteristics. In Model 2, number of sessions attended (β = 0.10, 95% CI = 0.07, 0.13, *p* < 0.001) and program mode (GBP: β = 0.14, 95% CI = 0.07, 0.21, *p* < 0.001) significantly influenced HLI scores though following adjustment for background socio-demographic factors (Model 3) mode was no longer significant (*p* = 0.076). Findings showed that being retired (β = 0.59, 95% CI = 0.51, 0.66, *p* < 0.001), aged 45 or older (β = 1.00, 95% CI = 0.90, 1.10, *p* < 0.001), and having a certificate or diploma (β = 0.32, 95% CI = 0.14, 0.50, *p* < 0.001) or bachelor’s degree or higher (β = 0.79, 95% CI = 0.61, 0.98, *p* < 0.001) conferred a higher average HLI while being male, Aboriginal or Torres Strait Islander background, or not currently working conferred lower average HLI scores (*p* < 0.001 for all).

To assess the changes of each health behaviour individually, the predicted probabilities for each health behaviour were estimated using nominal logistic models, with results showing consistent trends towards healthier lifestyle behaviours over the program period. Overall, dietary indices also showed a shift towards recommended dietary guidelines with 70% meeting the guidelines for daily fruit intake, 25% meeting the guidelines for daily vegetable intake, and 82% consuming sugar-sweetened drinks and take-away less than weekly.

**Fig. 1:**
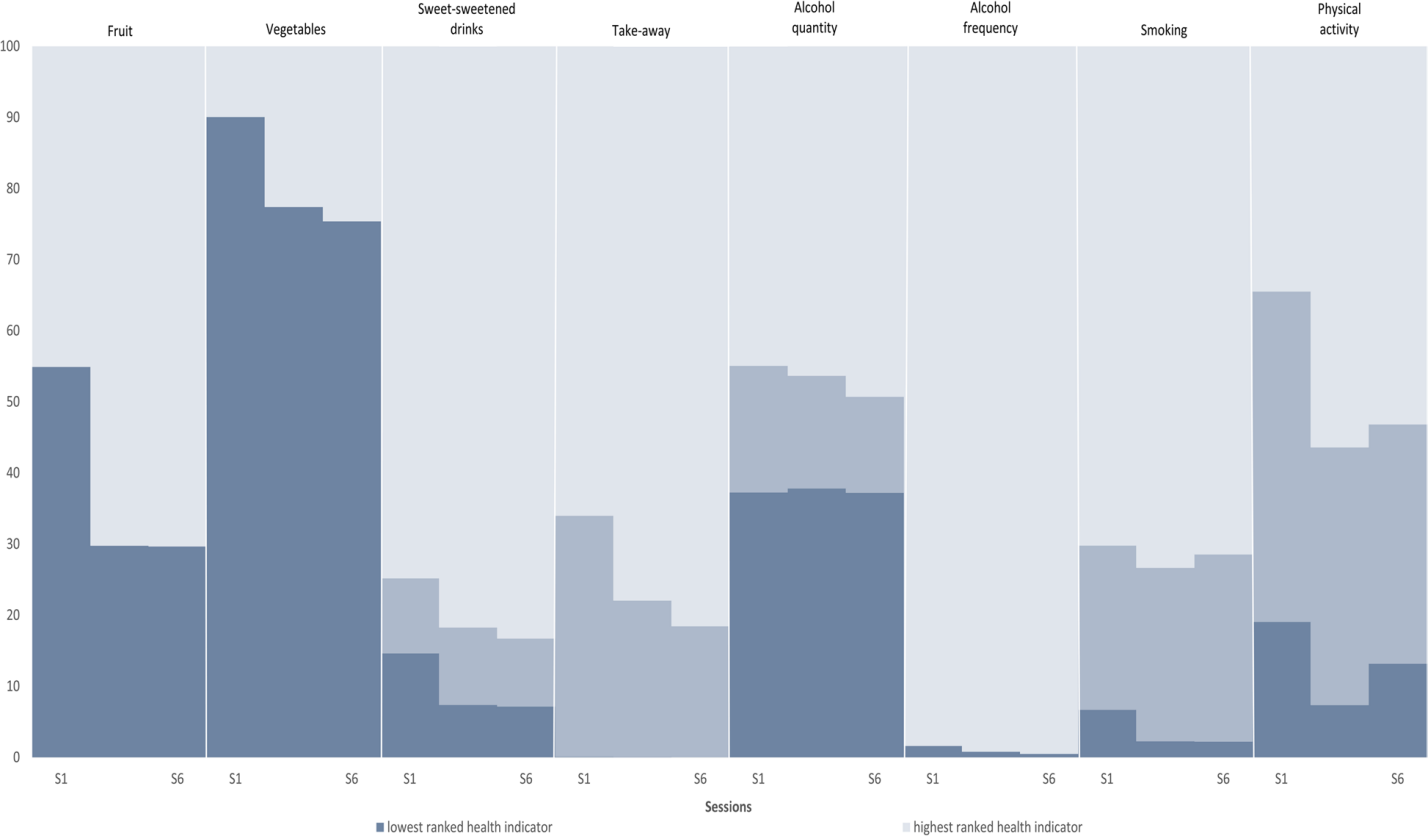
Lasagne plot for the predicted probabilities for individual health behaviours at Sessions 1, 5 and 6. Notes: (**a**) Fruit and vegetables (highest =  ≥ 2 serves fruit and ≥ 5 serves veg, lowest =  < 2 serves fruit and < 5 serves veg); (**b**) Sugar-sweetened beverages (highest =  < weekly; lowest =  > weekly); (**c**) Take-away (highest =  < weekly; lowest = daily); (**d**) Alcohol quantity (highest = none; highest =  ≥ 5 drinks per session); Alcohol frequency, (highest = daily; lowest = less than daily; (**e**) Smoking (highest = never smoked; lowest = current smoker); (**f**) Physical activity (highest = sufficiently active for health; lowest = sedentary); (**g**) The predicted probabilities from nominal logistic models were fitted separately for each health behaviour. Adjustment was made for study modality, no. sessions, time, employment status, sex, age bracket, educational attainment, First Nations People, and IRSAD quintile

Overall, few participants consumed alcohol daily (< 1%) though around one-third (37%) of participants consumed an average of 5 or more alcoholic drinks in one session and this was largely unchanged over the program period. Finally, at baseline 19% of participants reported being sedentary and 46% were insufficiently active for health. Over the program period, the proportion of people meeting physical activity guidelines increased, though at Session 6, only 53% reported being sufficiently active for health. Percentage changes using predicted probabilities in individual health behaviours from Session 1 to Session 6 are illustrated in Fig. 1.

## Discussion

This paper explores changes in primary health outcomes of participants from the *My health for life* program, which aimed to reduce the risk factors of chronic diseases among at-risk populations. When compared with the lifestyle indicators of Queenslanders more generally, *My health for life* participants reported lower compliance with recommended daily fruit consumption, higher baseline average single occasion risky drinking, and low physical activity levels that were sufficient for health [47]. Notably however, over the *My health for life* program period, the proportion of participants meeting recommended health behaviour guidelines (e.g., diet, smoking cessation, physical activity), in some instances, was greater than is reported by Queensland adults [47].

During the intervention, the proportion of participants in the extremely obese, obese, categories decreased from Session 1 to Session 6 while those in the normal weight range increased from 9 to 13%. Overweight and obesity is the fourth highest risk factor for burden of disease in Australia. A large proportion of total disease burden can be prevented avoiding or reducing exposure to risk factors including tobacco use, overweight (including obesity), dietary risks, and alcohol use. Overweight including obesity accounts for 8.4% of the burden of disease in Australia [48]. Obesity contributes 9.6% of all fatal burden and 7.4% of all non-fatal burden. Recent studies have shown that even modest reductions in BMI (~ 1 kg/m^2^) in ‘at-risk’ populations, is associated with a significant reduction in disease burden [2]. The downward trend in both BMI and waist circumference in the *My health for life* program participants has the potential to have a significant impact on the burden of chronic disease. The reduction in BMI for program participants is similar to previous literature which demonstrates the potential for programs targeting multiple health behaviours to contribute to reduction in BMI and waist circumference [8, 15]. This shows the value of targeting multiple modifiable risk factor behaviours in an intervention seeking to reduce the risk of chronic disease. Thus, improving modifiable health behaviours such as diet, smoking, physical activity, and risky alcohol consumption, especially before disease occurs, that is primary prevention, not only benefits the health and wellbeing of people, it also plays a role in controlling health care costs [3, 48].

Dietary indicators improved over time, with many participants increasingly likely to meet recommended fruit and vegetable intake at Session 5. In this study 73.5% of participants were meeting dietary guidelines for fruit consumption, whereas in Queensland, it is estimated that around 2.1 million (53%) adults were meeting recommendations for fruit consumption. Around one quarter (25.7%) of participants in this study were meeting the recommendations for vegetable consumption, compared to only 320,000 (8.0%) of Queensland adults meeting recommendations for vegetable consumption [47]. Importantly, these results are also higher than overall Australian adult levels of meeting recommendations for fruit consumption (48.5%) and vegetable consumption (7.5%). The favourable results demonstrated by this multiple health behaviour approach are consistent with existing research showing that optimal behaviour change occurs when addressing concurrent risk factors, rather than targeting unhealthy lifestyle behaviours individually [49]. Significant changes in other dietary indicators were noted over time. While greater than daily take-away consumption was low in this sample, weekly take-away meals were reported by around one-third of participants at baseline. Over the program period however, frequency of take-away intake was significantly reduced which, if maintained, might alter mortality risk. For example, a recent study of similarly aged participants (50–76 years) from Washington State in USA, showed highest fast-food intake (i.e., Quartile 4) conferred a ~ 16% increased risk of all-cause mortality compared lowest quartile of intake [50]. In Australia, 12% of men and 6% of women are likely to consume sugar-sweetened drinks daily [2], 16.3% of our sample were consuming sugar-sweetened drinks at least weekly at program commencement, this reduced to 8.2% by program end.

In 2019 in Australia there were 11.6% of the adult population who smoke tobacco daily [51], compared to our sample at baseline (8%) and dropping to 3.9% at Session 5 and 3.3% at session 6. Daily consumption of alcohol was higher in the Australian population (5.4%) [51] compared to our sample at baseline (2.2%) and at session 5 (0.9%) and session 6 (0.9%).

While smoking and alcohol consumption rates in this group were lower than the Australian population at baseline, there were improvements across the life of the program.

There were general improvements in participants’ physical activity behaviour between Session 1 (34.1%) and 5 (58%), though only around half of participants were sufficiently active for health at program completion, returning to lower levels (53.3%). At baseline for this study there was a considerably smaller percentage of participants who were sufficiently active for health (34.1%), than that previously reported for Queensland adults aged 18–75 years (59% completed the recommended minimum of 150 min of moderate intensity physical activity over at least five sessions in the previous week) and Australian adults more broadly (45%) [47, 52]. This shows potential for the program to improve physical activity to levels aligned to the general Australian population.

The results from this study are consistent with the growing body of research showing the effectiveness of lifestyle interventions on improving modifiable health behaviours. Key components of these interventions include the use of motivational interviews to allow participants to set their own intentions and goals [53, 54], structured programs which include education on multiple modifiable risk factor behaviours, group based programs [55], and underpinned by behaviour change theory [54]. Additional exploration of the environmental factors (e.g. safe spaces for physical activity) that can be manipulated to increase physical activity as well as the use of technologies (e.g. activity trackers) to enhance positive health behaviours might provide additional benefit to individuals for whom behaviour change is sub-optimal [56].

### Strengths and limitations

The intervention was associated with improvements in healthy lifestyle indicators which contributes new information to this research area. While participants showed improvements in dietary and smoking indicators, changes in alcohol consumption, and physical activity were less amenable to the program. Conducting additional research will help understand the multi-level barriers and facilitators of behaviour change in this context to further tailor the intervention for priority groups.

This paper has several limitations to acknowledge. First, a pragmatic non-randomised, time–series analysis adopting observational, goal-based, and pretest–posttest design features underpins the program evaluation. The absence of a control group, which would be extremely challenging to establish given the size and intent of the program, could impact on the ability to make causal inferences about the impact of the intervention of health indicators, however, where possible program results are compared to Queensland and Australian rates of behaviour. Participants enrolled in a lifestyle modification program, are potentially highly motivated to improve their health regardless of the intervention which may impact on the positive outcomes of the program. Second, akin with many longitudinal studies, missing data were present in around 42% of participants at Session 5 and 58% of participants at Session 6 and this might have impacted the magnitude of outcomes and the representativeness of the sample [37]. To account for missing data, patterns of missing data per assessed and as data were plausibly missing at random, multiple imputation were performed. In many instances data were collected by self-report, and despite efforts to minimise self-reporting bias through memory aids, multiple data-checkpoints and training of facilitators in data collection [57], there may have been increased risk of information bias which needs to be considered when viewing the validity of this research [58]. Finally, this study was conducted in one context, which may limit its generalisability, however there are many lessons that can be applied in other contexts including the need to include measures of multiple behaviours when seeking to reduce the risk of chronic disease in a population.

## Supplementary Information

**Additional file 1: Supplementary Table 1.** Healthy lifestyleindex construction. **Supplementary Table 2.** Sensitivity analysisof the longitudinal modelling of a HLI using Gaussian Generalized EstimatingEquations with an exchangeable structure and robust standard errors (with andwithout adjustment for baseline excess weight). **Supplementary Table 3. **Associations between participant characteristics and missing data at baseline. **SupplementaryTable 4.** Proportion of missing data from indicators at sessions 1-6.

## Data Availability

The data that support the findings of this study are available from Health and Wellbeing Queensland but restrictions apply to the availability of these data, which were used under license for the current study, and so are not publicly available. Data are however available from the authors upon reasonable request and with permission of Health and Wellbeing Queensland.

## Abbreviations

CVD: Cardiovascular disease
BMI: Body mass index
WC: Waist circumference
GEE: Generalized Estimating Equations
HLI: Healthy Lifestyle Index
IQR: Interquartile range
AUSDRISK: Australian Diabetes Risk assessment
CVD: Check Absolute Cardiovascular Disease Risk assessment
GBP: Group-based program
THC: Telephone health coaching programs
HAPA: Health Action Process Approach
CALD: Culturally or Linguistically Diverse
IRSAD: Index of Relative Socio-Economic Advantage and Disadvantage
QIC: Quasilikelihood under the Independence model Criterion

## Footnotes

1 While the impact of unhealthy lifestyle on chronic disease risk is not equivalent (e.g., 62% of coronary heart disease (CHD) and 41% of type 2 diabetes (T2D) is attributable to poor diet and 12% of CHD and 19% of T2D is attributable to physical inactivity [4]), this study aims to generally improve health behaviour. Thus, the simple additive method to compute a HLI was deemed appropriate.
