# Supplementary material for: Changes in health behaviours in adults at-risk of chronic disease: primary outcomes from the My health for life program
Source: BMC Public Health. 2022 Aug 30;22:1648. doi: 10.1186/s12889-022-14056-1 (PMC9429361; doi:10.1186/s12889-022-14056-1)
Supplement: Supplementary file 1 — Additional file 1: Supplementary Table 1. Healthy lifestyleindex construction. Supplementary Table 2. Sensitivity analysisof the longitudinal modelling of a HLI using Gaussian Generalized EstimatingEquations with an exchangeable structure and robust standard errors (with andwithout adjustment for baseline excess weight). Supplementary Table 3. Associations between participant characteristics and missing data at baseline. SupplementaryTable 4. Proportion of missing data from indicators at sessions 1-6. [file 12889_2022_14056_MOESM1_ESM.docx]

**Supplementary Table 1** Healthy lifestyle index construction

|  | Points per indicator | | |
| --- | --- | --- | --- |
|  | 2 | 1 | 0 |
| Daily fruit intake ^a^ |  | 2 or more serves | < 2 serves daily |
| Daily vegetable intake ^a^ |  | 5 or more serves | < 5 serves daily |
| Sugar sweetened drinks | Less than weekly | Weekly | Daily/Several times weekly |
| Take-away consumption | Less than weekly | Weekly | More than weekly |
| Alcohol quantity ^c^ | None | 1-4 drinks | ≥ 5 per session |
| Alcohol frequency ^c^ |  | Daily | Weekly or less |
| Smoking | Never | Past smoker | Current smoker |
| Physical activity ^b^ | Sufficient for health | Insufficient for health | Sedentary |

^a^ Current dietary guidelines recommend a minimum of 2 serves of fruit and 5 serves of vegetables per day [24]

^b^ Physical activity was defined according to the Australian Physical Activity Guidelines [25] denoting the accumulation of at least 150 minutes of activity over one week

^c^ Alcohol consumption was defined according to the Australian Health Survey [26] with ‘risky’ alcohol consumption defined as daily drinking or having more than 4 standard drinks on any one day

**Supplementary Table 2** Sensitivity analysis of the longitudinal modelling of a HLI using Gaussian Generalized Estimating Equations with an exchangeable structure and robust standard errors (with and without adjustment for baseline excess weight)

|  | Model 1^a^ | | Model 2^b^ | | Model 3^c^ | |
| --- | --- | --- | --- | --- | --- | --- |
|  | *β (95% CI)†* | *β (95% CI)‡* | *β (95% CI)†* | *β (95% CI)‡* | *β (95% CI)†* | *β (95% CI)‡* |
| Constant | 8.51 (8.46, 8.55)* | 8.35 (8.27, 8.43)* | 8.07 (7.97, 8.17)* | 7.97 (7.80, 8.13)* | 6.85 (6.63, 7.08)* | 6.76 (6.51, 7.02)* |
| Sessions |  |  |  |  |  |  |
| Session 1 | - |  | - | - | - | - |
| Session 5 | 0.97 (0.90, 1.03)* | 0.85 (0.77, 0.94)* | 0.96 (0.89, 1.03)* | 0.81 (0.72, 0.90)* | 0.98 (0.91, 1.05)* | 0.82 (0.73, 0.91)* |
| Session 6 | 1.20 (1.13, 1.27)* | 1.22 (1.13, 1.31)* | 1.19 (1.12, 1.27)* | 1.18 (1.08, 1.27)* | 1.20 (1.13, 1.28)* | 1.17 (1.08, 1.26)* |

^a^ Model 1, unadjusted relationship between HLI and time (sessions 5 and 6)

^b^ Model 2, adjusted for program characteristics (delivery mode and no. sessions attended)

^c^ Model 3, adjusted for program characteristics and personal background (employment status, sex, age bracket, educational attainment, First Nations People, and IRSAD quintile)

† Original model

‡ Model adjusted for BMI and WC

* *p* <.01

**Supplementary Table 3** Associations between participant characteristics and missing data at baseline

|  | HLI | |
| --- | --- | --- |
|  | Full | Partial |
|  | n (%) | n (%) |
| Mode |  |  |
| THC | 3,339 (35.6) | 57 (6.3)* |
| GBP | 6,033 (64.4) | 846 (93.7) |
| Employment statis |  |  |
| Employed | 4,847 (54.1) | 364 (43.9)* |
| Home duties | 428 (4.8) | 64 (7.7) |
| Retired | 2,497 (27.9) | 282 (34.0) |
| Not working | 627 (7.0) | 51 (6.2) |
| Other | 554 (6.2) | 68 (8.2) |
| Sex |  |  |
| Female | 7,206 (77.3) | 671 (76.6) |
| Male | 2,112 (22.7) | 205 (23.4) |
| Age bracket |  |  |
| <45 years | 1,362 (14.6) | 132 (14.8) |
| 45 or older | 7,989 (85.4) | 758 (85.2) |
| ATSI |  |  |
| No | 8,985 (95.9) | 872 (96.6) |
| Yes | 387 (4.1) | 31 (3.4) |
| Educational attainment |  |  |
| Primary education | 312 (3.4) | 43 (5.2)* |
| Secondary education | 2,760 (30.1) | 295 (35.4) |
| Certificate/diploma | 3,326 (36.2) | 296 (35.5) |
| Bachelor/postgraduate | 2,643 (28.8) | 174 (20.9) |
| Other | 141 (1.5) | 26 (3.1) |
| CALD |  |  |
| No | 9,096 (97.1) | 876 (97.0) |
| Yes | 276 (2.9) | 27 (3.0) |
| IRSAD quintile |  |  |
| Quintile 1 | 1,218 (13.0) | 169 (18.7)* |
| Quintile 2 | 1,525 (16.3) | 142 (15.7) |
| Quintile 3 | 2,136 (22.8) | 193 (21.4) |
| Quintile 4 | 2,090 (22.3) | 215 (23.8) |
| Quintile 5 | 2,395 (25.6) | 184 (20.4) |
| General health |  |  |
| Fair/poor | 3,676 (39.7) | 356 (42.2) |
| Excellent/good | 5,587 (60.3) | 488 (57.8) |

HLI, Healthy life index (comprising dietary, physical activity, alcohol and smoking indices); THC, Telephone health couching; GBP, Group-based program; CALD, Culturally or Linguistically Diverse

* *p* < .01

**Supplementary Table 4** Proportion of missing data from indicators at sessions 1-6 ^a^

|  | Session 1 | Session 5 | Session 6 |
| --- | --- | --- | --- |
| No. missing data points | n (%) | n (%) | n (%) |
| None | 9,372 (91.2) | 5,965 (58.1) | 4,295 (41.8) |
| 1 | 869 (8.5) | 473 (4.6) | 268 (2.6) |
| 2 | 33 (0.3) | 264 (2.6) | 878 (8.5) |
| 3 | 1 (0.0) | 12 (0.1) | 5 (0.0) |
| 4 |  | 1 (0.0) | 2 (0.0) |
| 5 |  | 1 (0.0) | 4 (0.0) |
| 6 |  | 5 (0.0) | 158 (1.5) |
| 7 |  | 3,093 (30.1) | 4,194 (40.8) |
| 8 |  | 461 (4.5) | 471 (4.6) |

^a^ Indicators included her include dietary, physical activity, and alcohol and tobacco (range 0-13)
